# Development of machine learning models for predicting non-remission in early RA highlights the robust predictive importance of the RAID score-evidence from the ARCTIC study

**DOI:** 10.3389/fmed.2025.1526708

**Published:** 2025-02-12

**Authors:** Gaoyang Li, Shrikant S. Kolan, Franco Grimolizzi, Joseph Sexton, Giulia Malachin, Guro Goll, Tore K. Kvien, Nina Paulshus Sundlisæter, Manuela Zucknick, Siri Lillegraven, Espen A. Haavardsholm, Bjørn Steen Skålhegg

**Affiliations:** ^1^Division of Molecular Nutrition, Department of Nutrition, Institute of Basic Medical Sciences, University of Oslo, Oslo, Norway; ^2^Center for Treatment of Rheumatic and Musculoskeletal Diseases (REMEDY), Division of Rheumatology and Research, Diakonhjemmet Hospital, Oslo, Norway; ^3^Institute of Clinical Medicine, University of Oslo, Oslo, Norway; ^4^Oslo Centre for Biostatistics and Epidemiology, Department of Biostatistics, Institute of Basic Medical Sciences, University of Oslo, Oslo, Norway

**Keywords:** rheumatoid arthritis, methotrexate, remission, prediction, machine learning

## Abstract

**Introduction:**

Achieving remission is a critical therapeutic goal in the management of rheumatoid arthritis (RA). Despite methotrexate being the cornerstone of early RA treatment, a significant proportion of patients fail to achieve remission. This study aims to predict 6-month non-remission in 222 disease-modifying anti-rheumatic drug (DMARD)-naïve RA patients initiating methotrexate monotherapy, using baseline patient characteristics from the ARCTIC trial.

**Methods:**

Machine learning models were developed utilizing twenty-one baseline demographic, clinical and laboratory features to predict non-remission according to ACR/EULAR Boolean, SDAI and CDAI criteria. The model employed a super learner algorithm that combine three base algorithms of elastic net, random forest and support vector machine. The model performance was evaluated through five independent unseen tests with nested 5-fold cross-validation. The predictive power of each feature was assessed using a composite measure derived from individual algorithm estimates.

**Results:**

The model demonstrated a mean AUC-ROC of 0.75-0.76, with mean sensitivity of 0.77-0.81, precision (also referred to as Positive Predictive Value) of 0.77-0.79 and specificity of 0.63-0.66 across the criteria. Predictive power analysis of each feature identified the baseline Rheumatoid Arthritis Impact of Disease (RAID) score as the strongest predictor of non-remission. A simplified model using RAID score alone demonstrated comparable performance to the full-feature model.

**Conclusion:**

These findings highlight the potential utility of baseline RAID score-based model as an effective tool for early identification of patients at risk of non-remission in clinical practise.

## Highlights

- Machine learning models effectively predict non-remission in early RA patients on methotrexate monotherapy.
- High baseline RAID scores strongly predict non-remission, underscoring its role in RA management.
- A RAID-focused model offers comparable predictive power to full-feature model, streamlining clinical assessments.

## Introduction

There is currently no curative treatment available for rheumatoid arthritis (RA), and the treatment goals are to minimize inflammation, prevent structural joint damage and maintain physical function (1, 2). Methotrexate, a conventional synthetic disease-modifying anti-rheumatic drug (csDMARD) that interferes with B vitamin folate metabolism, is the cornerstone of RA management and widely accepted as the first-line treatment for RA patients (3). To optimize RA management, the treat-to-target strategy, which involves regular monitoring and adjusting treatments to achieve and maintain specific clinical targets, has been widely adopted in clinical settings (3, 4). The optimal treatment target is to attain long-term remission (3), however in a significant proportion of patients, ranging from 50 to 60%, this is not achieved using methotrexate monotherapy (5–8). Early identification of patients unlikely to achieve remission could allow for tailored treatment strategies with clinical benefits.

Machine learning-based approaches have facilitated the integration of complex, heterogeneous and multi-dimensional datasets in prediction models. Algorithms such as elastic net (EN), random forest classifier (RFC) and support vector machine (SVM) have been demonstrated as effective in forecasting drug responsiveness (9–12), including the prediction of methotrexate effectiveness in RA patients (13–15). These algorithms, when integrated through ensemble learning methods such as the super learner algorithm, provide robust and generalizable performance, and have been accordingly employed in our predictive modeling (16). Recently, machine learning models have identified various baseline characteristics in predicting methotrexate response. These include biological markers such as anti-citrullinated protein antibodies (ACPA) (17), clinical characteristics such as disease activity score (DAS) (15, 17, 18), as well as treatment-related factors like corticosteroid co-treatment (15). Despite these insights, the predictive value of these baseline characteristics for non-remission following methotrexate treatment in RA remain underexplored. In an approach to facilitate personalized therapy for DMARD-naive RA patients, we utilized baseline characteristics from 222 DMARD-naïve patients with RA from the ARCTIC trial to develop machine learning models capable of predicting 6-month non-remission as per ACR/EULAR Boolean, SDAI, and CDAI criteria (19). The study aims to assess the predictive performance of these models across various non-remission criteria and identify key predictors at treatment onset for non-remission at 6 months.

## Methods

### Study population

Among the 230 patients enrolled in the ARCTIC trial, 224 individuals with available blood samples were approved for data access by the Norwegian regional ethics committee. Two patients were excluded due to insufficient sample quality, resulting in a final study population of 222 patients. This secondary analysis of an existing dataset fixed the sample size by the original trial design and data availability (19). All patients fulfilled the American College of Rheumatology/The European Alliance of Associations for Rheumatology (ACR/EULAR) 2010 classification criteria for RA, with symptom duration less than two years. Patients were DMARD naïve with indication to start methotrexate therapy and were randomized 1:1 to a treat-to-target strategy with or without the guidance of ultrasound examinations. Results from the primary analyses of the ARCTIC trial found no significant differences in clinical and radiographic outcomes between the two groups, and for this report the two groups were pooled. Methotrexate monotherapy was initiated combined with bridging therapy with tapering doses of prednisolone from 15 mg/day to 0 over 7 weeks (see details in the previous study) (19). The present study was conducted in compliance with the Declaration of Helsinki and was approved by the regional ethics committee (reference number: 2010/744/REK sør-øst C). All patients provided written informed consent. Patients, funders, or the public were not involved in the design, conduct, or reporting of our research.

### Baseline predictors and outcomes

The candidate predictors were all assessed at baseline (19). Demographic features included age, body mass index (BMI), sex, history of smoking. Main clinical and laboratory measures included time since patient-reported first swollen joint (symptom duration), rheumatoid factor (RF), anti-citrullinated protein antibodies (ACPA), erythrocyte sedimentation rate (ESR), C-reactive protein (CRP), swollen joint count based on 44 joints (SJC44), tender joint count using Ritchie Articular Index (RAI), Disease Activity Score (DAS), Clinical Disease Activity Index (CDAI) (20), Simplified Disease Activity Index (SDAI) (21), Rheumatoid Arthritis Impact of Disease total score (RAID score) (22), patient global assessment of disease activity (PGA), Physician global assessment of disease activity (PhGA), fatigue, joint pain, the patient-reported outcomes measurement information system physical function, T-score (PROMIS-PF), and finally, dose of methotrexate (see Supplementary Table S1 for details).

Outcomes were non-remission at 6 months assessed across three different criteria: ACR/EULAR Boolean, SDAI and CDAI. The ACR-EULAR Boolean criteria is based on Boolean statuses of four parameters (tender joint count, swollen joint count, CRP and PGA), serving as a stringent measure for assessing remission status. By contrast, SDAI and CDAI criteria employ a composite score comprising tender joint count, swollen joint count, PGA and PhGA, with the former also including CRP (see Supplementary Table S2 for details). Patients who transitioned from methotrexate monotherapy at the evaluated timepoint due to inadequate efficacy or intolerance, were classified as non-remission. Detailed statistics on the characteristics of patients categorized as remission or non-remission based on these criteria are presented in Supplementary Table S3.

### Data cleaning and imputation

Among all baseline measures, six had one or two data points missing from subjects (Supplementary Table S1). To prevent data leakage, missing values in these features were imputed separately for the training and test sets using the KNN Imputer from the Scikit-learn library. The KNN Imputer, a single imputation method, was chosen for its ability to estimate missing values based on feature similarity. Given the low proportion of missing data (see Supplementary Table S1), the potential bias introduced by imputation is considered minimal. Additionally, missing outcomes according to ACR/EULAR Boolean, SDAI and CDAI non-remission, which ranges from 12 to 13 instances, were imputed through a detailed analysis that consider DAS scores and data from follow-up visits at various time points (for details see Supplementary Data S1). All imputation procedures were conducted by a module named impute in Scikit-learn in Python 3.8.0 (23).

### Establishment of machine learning models

**Figure 1:**
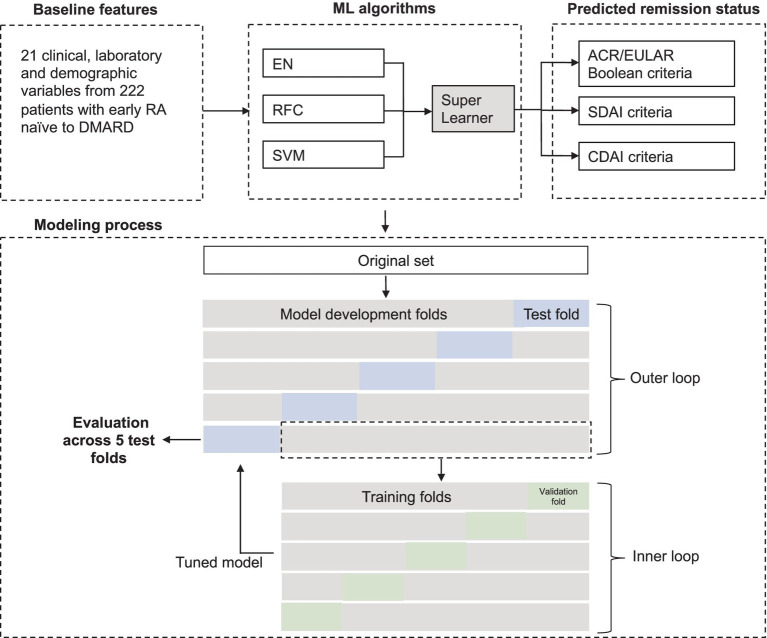
Analytical framework for predictive model development. Baseline features were used to predict 6-month non-remission via a super learner approach integrating three algorithms: EN, RFC, and SVM. A 5-fold nested cross-validation (CV) was utilized, with the outer loop partitioning the dataset into five folds, each sequentially serving as a hold-out test set. The remaining four folds were used for model development through a secondary 5-fold CV in the inner loop. Model performance was assessed across five test folds and displayed as mean (standard deviation). RA, Rheumatoid Arthritis; ML, Machine learning.

To construct machine learning models that integrate all baseline features, all instances were included to predict non-remission. For the simplified model, instances with missing features were excluded for the analysis, resulting in 220 patients for the RAID score-exclusive model. The data were split into two parts: 80% for training model (model development folds) and 20% for testing the model (test fold), as part of the nested 5-fold cross-validation detailed in Figure 1. Categorical features were converted into dummy variables indicating the absence or presence of a category by 0 or 1, and continuous features were standardized separately for training and test data to prevent data leakage. A super learner model was employed, integrating EN, RFC and SVM as base model and logistic regression as meta model. The training process involved two steps: (1) tuning optimal hyperparameter for each base model using 5-fold cross validation with grid search strategy (detailed in Supplementary Table S4; Supplementary Table S2), (2) initializing base models with optimal hyperparameters and generating an ensemble prediction through a weighted combination of predictions from base prediction algorithms via meta-algorithm. The model outputs a probability of non-remission, and final decision threshold was determined to maximize sensitivity while maintaining specificity at 0.60. All procedures were executed by modules (ensemble, metrics, model_selection, linear_model, svm) in Scikit-learn in Python 3.8.0 (23).

### Performance evaluation of machine learning models

Developing machine learning models requires training data on the training set and evaluation on an independent set from the training set to avoid data leakage and model overfitting. Nested cross-validation was preferred to avoid overfitting during model development. In this case, nested 5-fold cross-validation included two layers of cross-validation. The outer layer yielded 5 independent test sets and model development sets, allowing 5 sequential evaluations on test sets unseen by the model (Figure 1). The mean (SD) of these 5 test sets was used for analysis. The evaluation was performed with a minimum of three repetitions to ensure robustness, and the repetition with median performance was selected for presentation. AUC-ROC, sensitivity, specificity and precision were used as evaluation metrics. AUC-ROC measures the model’s ability to distinguish between classes, with a value of 1 indicating perfect separation. Sensitivity quantifies the proportion of true positives among actual positives, while specificity represents the proportion of true negatives among actual negatives. Precision, also known as Positive Predictive Value or PPV, refers to the proportion of true positives among all predicted positives (true positives plus false positives). The classification threshold was selected to maximize the sensitivity while maintaining specificity of at least 0.60. All procedures were executed by NumPy and Pandas libraries, and modules (ensemble, metrics, model_selection, linear_model, svm) in Scikit-learn in Python 3.8.0 (23).

### Feature predictive power interpretation

The predictive power of each feature was evaluated by a composite measure that integrates value of coefficient estimated from EN, feature important from RFC and permutation importance from SVM. All values were normalized using min-max normalization to eliminate the influence of scale discrepancies and ensure a uniform basis for comparison and interpretation. These values were then combined with weights estimated for each base learner by the meta learner. The directionality of association between each feature and outcomes was denoted by the directionality of coefficients from EN. All procedures were conducted by NumPy and Pandas libraries, and modules (ensemble, linear_model, inspection, preprocessing) in Scikit-learn library in Python 3.8.0 (23).

### Metric curve plot for interpreting clinical significance

Mean curve for sensitivity, precision and specificity across different thresholds were visualized from evaluations on 5 independent test sets using an interpolation method. In a clinically simulated context, the mean optimal threshold is set when precision reaches at least 0.90 in the mean curve. All procedures were conducted by NumPy, Matplotlib, Plotly libraries in Python 3.8.0 (23).

## Results

### Machine learning models establishment and evaluation framework

Machine learning models incorporating 21 baseline characteristics from 222 DMARD-naïve patients with early RA, as part of the ARCTIC study, were developed to target 6-month non-remission based on three criteria: (1) ACR/EULAR Boolean, (2) SDAI, and (3) CDAI criteria (Figure 1; Supplementary Table S2). The non-remission proportions observed were 64% (*n* = 143) for ACR/EULAR Boolean, 60% (*n* = 134) for SDAI, and 61% (*n* = 135) for CDAI. Baseline candidate predictors included patient demographics, laboratory results as well as clinical assessments, detailed in Supplementary Table S1. Among the categorical predictors, 62% of patients were females (*n* = 137), 82% tested positive for ACPA (*n* = 182), and 71% were RF positivity (*n* = 157). The median DAS at baseline was 3.3, with an interquartile range of 2.6 to 4.2. Additional continuous predictors were summarized in Supplementary Table S1. To build the model, a super learner algorithm was implemented, which integrates three base algorithms - EN, RFC and SVM – in a weighted manner which determined by meta learner algorithm of logistic regression. The model training and evaluation process employed a nested 5-fold cross-validation, where prediction performance evaluations were iterated across five independent unseen test set, with the remaining data used for model development (Figure 1).

### Prediction models incorporating comprehensive baseline characteristics exhibit promising performance in predicting non-remission at 6 months

**Figure 2:**
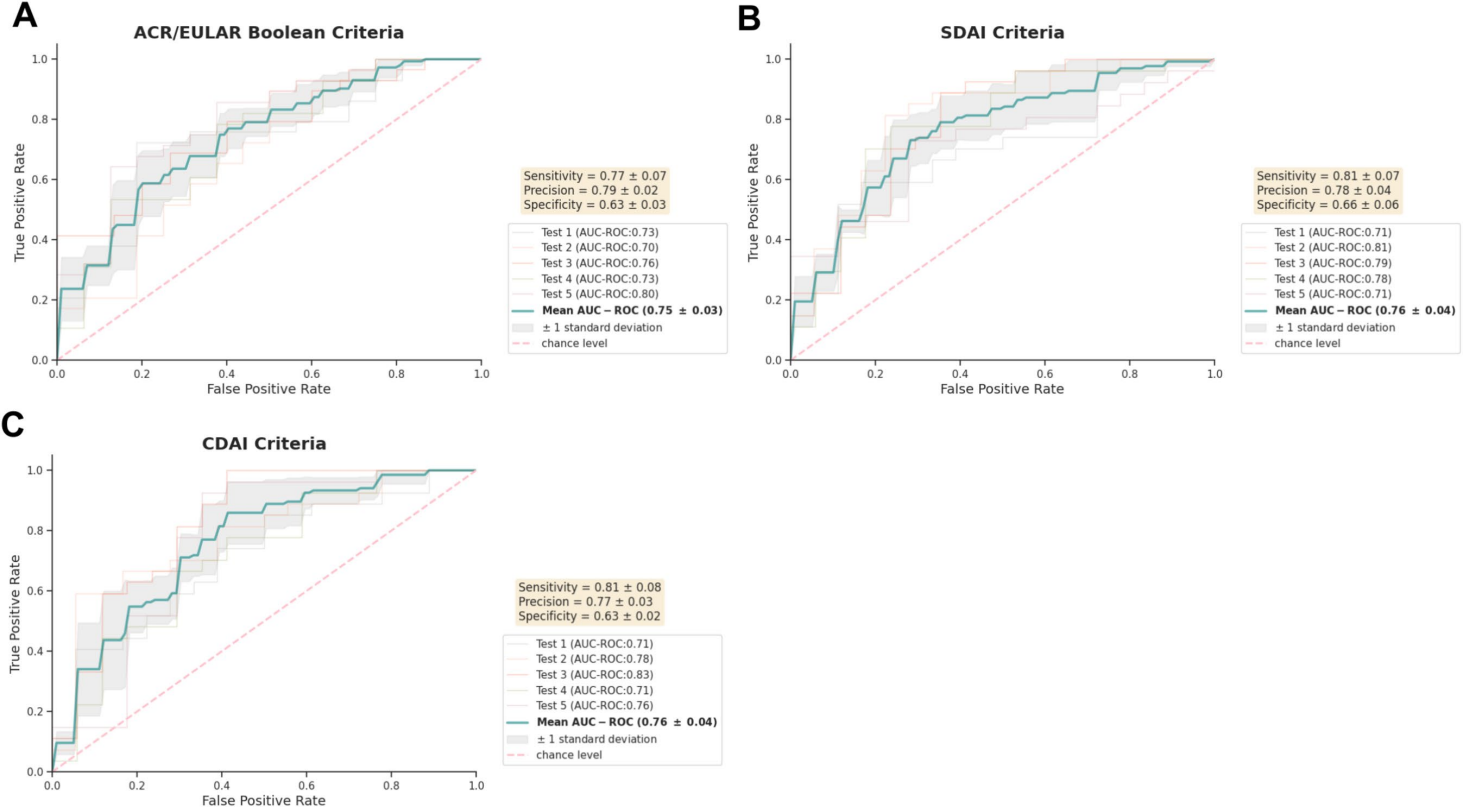
Performance of predictive models for non-remission post methotrexate monotherapy in early DMARD-naïve RA patients. AUC-ROC curves of models using baseline demographic, clinical and laboratory characteristics are shown for predicting 6-month non-remission according to **(A)** ACR/EULAR-, **(B)** SDAI-, **(C)** CDAI-criteria. Performance was assessed on 5 hold-out test sets (*n* = 44-45) through 5-fold nested cross-validation, reported as mean (standard deviation). Five repetitions of evaluation were implemented, with the median result presented. The classification threshold maximized sensitivity while maintaining a minimum specificity of 0.60. Precision also refers to as Positive Predictive Value. AUC-ROC, the area under the receiver operating characteristic curve.

The predictive ability for non-remission according to ACR/EULAR Boolean, SDAI and CDAI criteria was evaluated using mean AUC-ROC calculated from evaluations on five independent unseen test sets, achieving scores of 0.75, 0.76 and 0.76, respectively, (Figure 2). These representative evaluation results reflect the median value from five different repetitions presented in Supplementary Table S5. All repetitions reveal comparable AUC-ROC performance, with ranges of 0.73–0.77, 0.73–0.77, and 0.75–0.77 for ACR/EULAR Boolean, SDAI and CDAI criteria, respectively (Supplementary Table S5). Based on the representative result, we further assess the performance through mean sensitivity, precision, and specificity across the five test sets. Considering the clinical significance of identifying patients in non-remission, we set a threshold to achieve optimal sensitivity while maintaining decent specificity of at least 0.60. With such threshold, sensitivity ranged from 0.77-0.81, indicating that up to 81% of non-remission cases at 6 months can be correctly distinguished by the model, while precision reached 0.77-0.79, revealing the accuracy reached nearly 80% of predicted non-remission cases by the model (Figure 2).

### Rheumatoid arthritis impact of disease (RAID) score at the baseline was identified as a robust predictor for non-remission at 6 months

**Figure 3:**
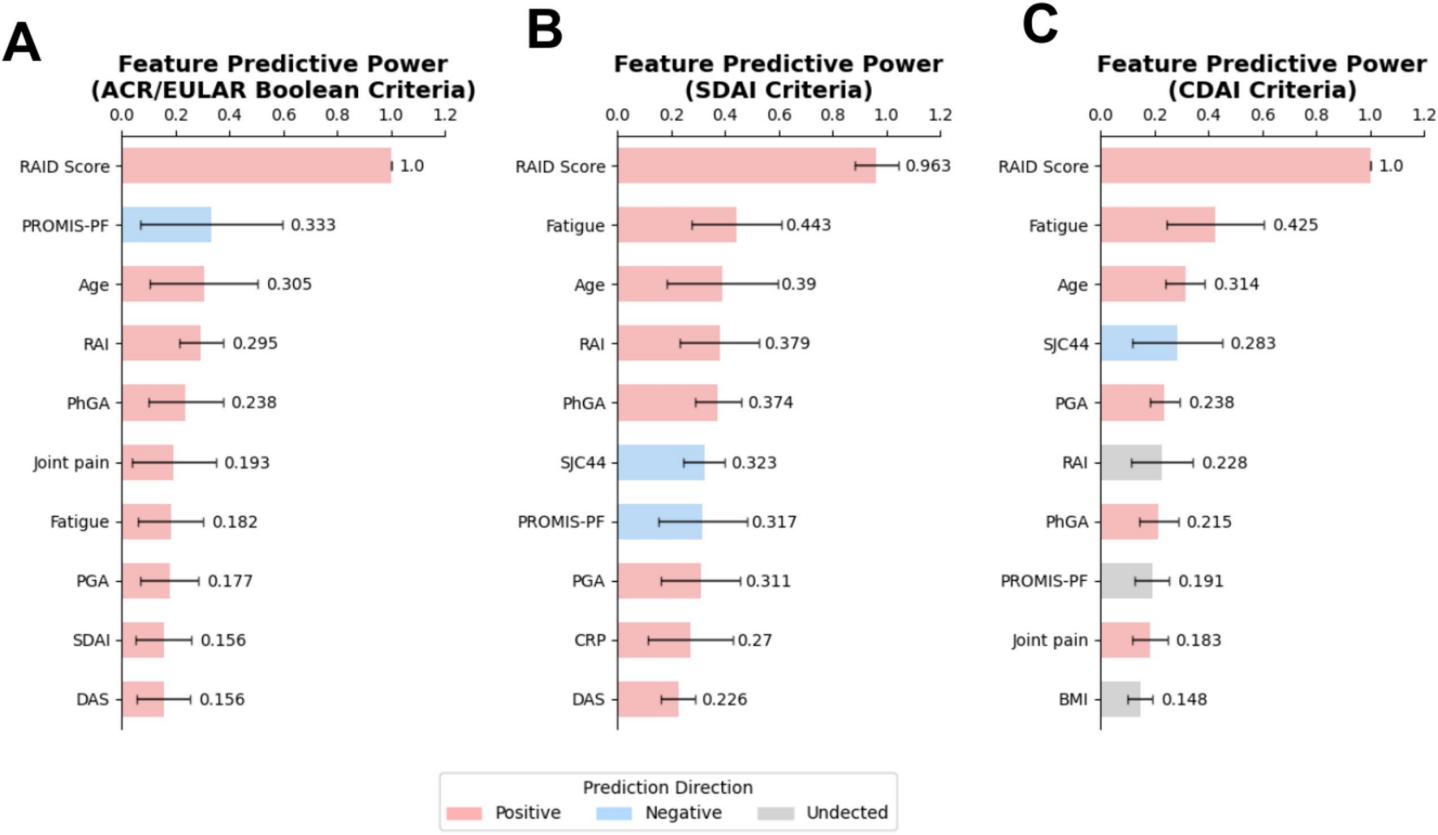
RAID score displayed an outstanding predictive power for predicting non-remission. Top-ranked baseline characteristics were identified in models predicting non-remission based on **(A)** ACR/EULAR Boolean-, **(B)** SDAI-, **(C)** CDAI-criteria. Feature predictive power in the super learner model was assessed using a composite measure that integrates coefficient value, impurity importance and permutation importance, respectively, estimated from three base models of super learner algorithm: EN, RFC, and SVM. Feature directionality was inferred from EN model coefficients, with undetected features noted as such (see Methods section for details). EN, Elastic Net; RFC, Random Forest Classifier; SVM, Support Vector Machine.

Next, we evaluated the predictive power of each feature by calculating a composite measure that integrates coefficient estimated from EN, feature important from RFC and permutation importance from SVM in a weighted and normalized manner. The direction of the association between features and outcomes was identified using coefficients from EN model. The top-ten ranking plot shown in Figure 3 reveals that RAID score, a patient-derived score evaluating the impact of RA on several domains of health including pain, functional disability, fatigue, sleep disturbances, coping, physical well-being, and emotional well-being, emerged as the top-ranked predictor for all non-remission outcomes, significantly outperforming other features. Following RAID score, PROMIS-PF ranked second for ACR/EULAR Boolean based non-remission, with a negative association with non-remission. Fatigue ranked just after RAID score for SDAI and CDAI based non-remission. Age, RAI, PhGA and PGA were also identified as important predictors across all three outcomes, although they exhibited lower importance, with importance scores ranging from 0.390-0.177 (Figure 3).

### Development and evaluation of simplified prediction models using a single robust predictor

**Figure 4:**
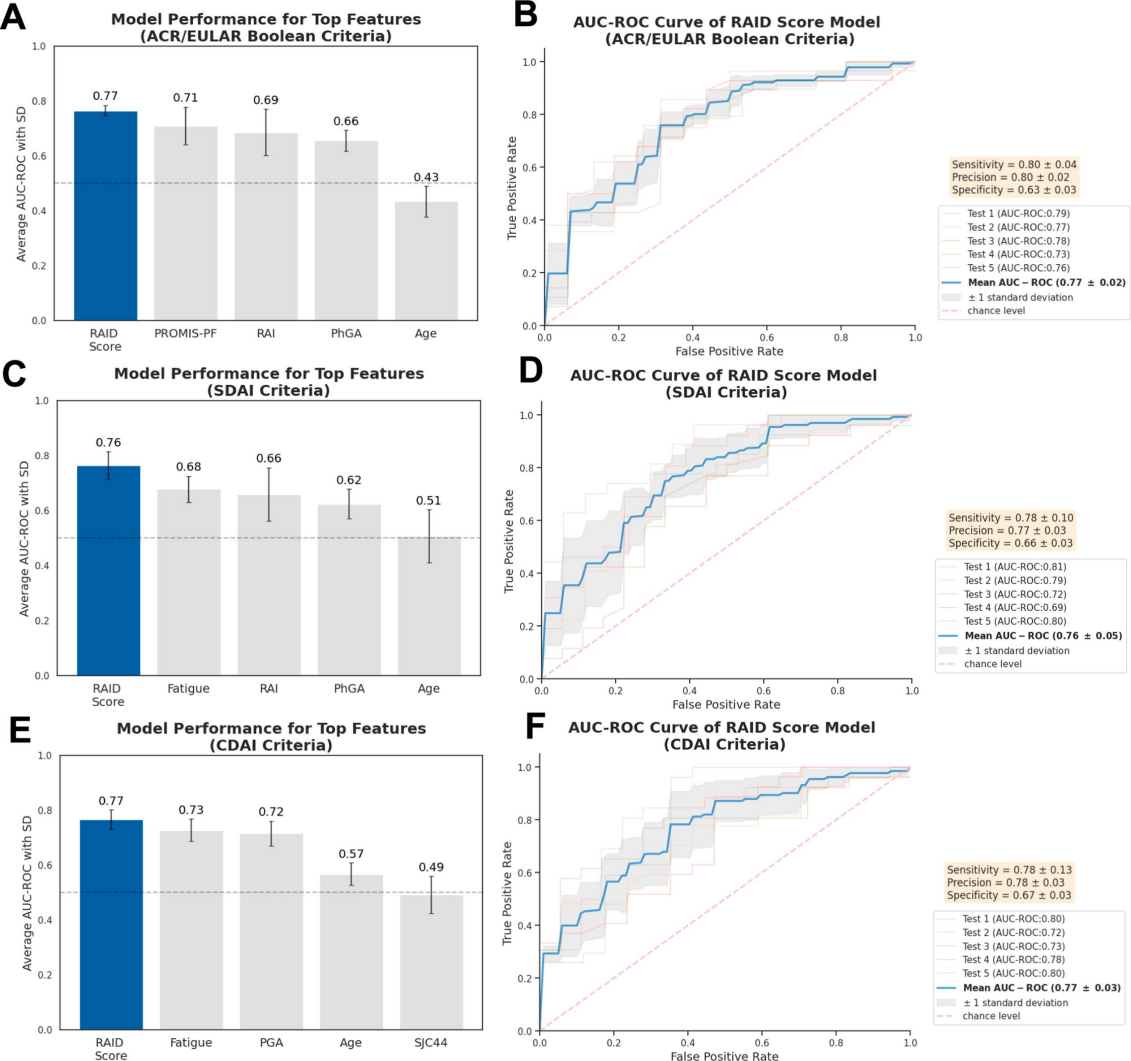
Performance of simplified models using individual features. AUC-ROC values are shown for models predicting non-remission using single baseline characteristic according to **(A)** ACR/EULAR-, (**C**) SDAI-, **(E)** CDAI-criteria, focusing on top five predictive features indicated earlier. The best-performing models are illustrated by AUC-ROC curve for non-remission outcomes **(B,D,F)**. Evaluation was performed across five independent hold-out test sets (*n* = 44) using nested 5-fold cross-validation, with results presented as mean (standard deviation). Three repetitions were performed, with the one achieving median value for RAID score-based model presented. Precision also refers to as Positive Predictive Value. AUC-ROC, the area under the receiver operating characteristic curve.

To further substantiate the significance of robust predictors, and to explore the utility of a simplified model, individual models were developed using each of the top five identified predictors as the sole feature. The analysis indicated that models relying solely on the RAID score consistently outperformed those based on other single predictors for predicting non-remission across various criteria, with the mean AUC-ROC of 0.76–0.77, mean sensitivity of 0.78–0.80, mean precision of 0.77–0.80 and mean specificity of 0.63–0.67. This performance is comparable to that of the comprehensive model incorporating all baseline features, affirming the critical role of the RAID score in predicting non-remission (Figure 4). Consistent with the findings presented in Figure 3, models based on the second-ranked predictors, PROMIS-PF and fatigue, also demonstrated strong performance for predicting ACR/EULAR Boolean and SDAI/CDAI-based non-remission respectively, achieving AUC-ROC of 0.71 and 0.68-0.73. Notably, despite not ranked top by composite predictive power analysis, the PGA exhibited notable predictive efficacy for CDAI non-remission with AUC-ROC of 0.72. In contrast, age displayed limited performance across all non-remission predictions, despite its modest predictive power rankings as identified in Figure 3.

### Further model performance evaluation of RAID score-exclusive model

**Figure 5:**
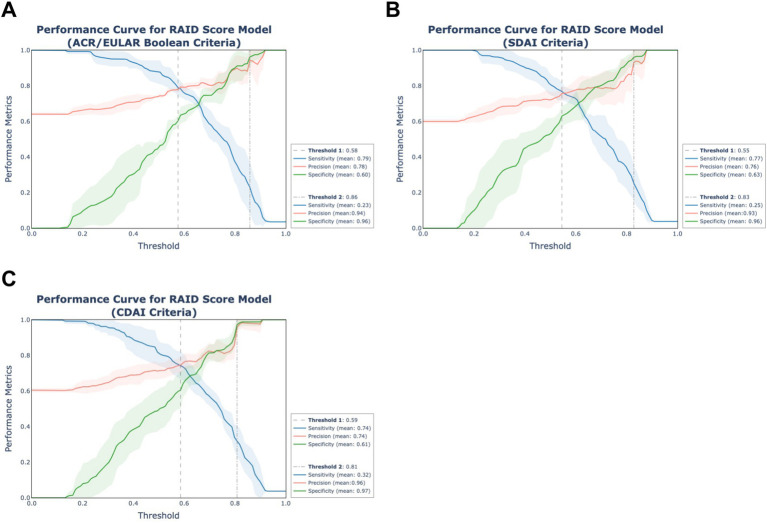
Clinical significance of simplified RAID score models. The curves of sensitivity, precision and specificity across thresholds of the simplified models using RAID score alone were displayed for remission status according to **(A)** ACR/EULAR-, **(B)** SDAI-, **(C)** CDAI-criteria. The evaluation was performed in five test sets using a nested 5-fold cross-validation approach with results represented as solid lines (mean) and shaded areas (standard deviation) derived through interpolation. The dashed line (Threshold 1) represents the classification threshold maximizing sensitivity while maintaining a minimum specificity of 0.60. The dot-dashed line (Threshold 2) denotes the threshold achieving a minimum precision of 0.90. Precision also refers to as Positive Predictive Value.

The RAID score-exclusive model demonstrates considerable potential for clinical application due to its accessibility, simplicity, and notable performance metrics. To assess its clinical utility more thoroughly, we analyzed the dynamic patterns of sensitivity, specificity and precision across various thresholds. These thresholds were used to classify patients into remission and non-remission categories at 6 months. This analysis was conducted on five independent test sets not previously seen during model training. Interpolation methods were employed to compute mean performance curve across these evaluations, as depicted in Figure 5. Two classification thresholds were defined for distinct clinical objectives within a simulated clinical context. The first threshold prioritized the identification of non-remission cases by maximizing true positives. For consistency, we retained a previously defined threshold that maximized sensitivity while maintaining acceptable specificity (>0.60). The thresholds determined for non-remission were 0.58, 0.55, and 0.59 for ACR/EULAR Boolean, SDAI, and CDAI criteria, respectively, yielding mean sensitivity values of 0.79, 0.77, and 0.74, with corresponding mean precision values of 0.78, 0.76, and 0.74. The second threshold aimed to minimize false positives, which is critical in scenarios requiring high precision for accurate non-remission predictions. To this end, the classification threshold was adjusted to achieve a mean precision of at least 0.90, corresponding to a false positive rate of less than 10%. We set classification thresholds of 0.86, 0.83, and 0.81 for non-remission based on the ACR/EULAR Boolean, SDAI, and CDAI criteria, respectively, in this case. These thresholds produced mean sensitivity values of 0.23, 0.25, and 0.32, as well as mean precision values of 0.94, 0.93, and 0.96, respectively.

Further, we investigated the relationship between RAID score and predicted probability through scatter plot analysis. This analysis revealed that as RAID score increased, the range of predicted probabilities narrowed, indicating more consistent prediction outcomes for patients with higher RAID scores (Supplementary Figure S1). According to the thresholds identified in Figure 5, 32 patients were classified as non-remission under both ACR/EULAR Boolean and SDAI criteria, and 41 under CDAI criteria. Among these population, misclassifications occurred for 3, 3, and 2 patients, respectively. The minimum RAID score at the baseline for patients predicted to be in non-remission were 6.0, 6.6, and 5.7, respectively.

## Discussion

Despite the role of methotrexate as a cornerstone therapy in early RA, the heterogeneity in patient response to this treatment remains a significant challenge. This underscores a pressing need to enable personalized treatment approaches and the early identification of patients who are unlikely to achieve disease remission using methotrexate monotherapy alone. Although various prediction models have been development, comprehensive exploration of accessible baseline demographic, clinical and laboratory predictors for outcomes across diverse remission criteria remains insufficiently studied. Here, we investigated the potential to predict non-remission across three criteria: ACR/EULAR Boolean, SDAI and CDAI in DMARD-naïve RA patients undergoing methotrexate monotherapy and managed with a treat-to-target strategy. Employing machine learning and nested cross-validation evaluation, we found that models incorporating all baseline features achieved robust predictive performance with an AUC-ROC of 0.75 to 0.76 for non-remission at 6 months. The RAID score emerged as a reliable and robust predictor. The simplified model based solely on the baseline RAID score demonstrated performance comparable to more complex models, underscoring its potential in guiding methotrexate therapy decisions.

Our study demonstrated high predictive performance across the three non-remission criteria, highlighting the model’s robustness across both Boolean and composite scores. This promising performance reveals predictive value of baseline patient characteristics, aligning with other studies that have successfully utilized patient characteristics to predict clinical outcomes like low-disease activity or DAS-based EULAR response criteria (15, 17).

A significant finding from our study is the RAID score’s powerful predictive capability for non-remission. As a composite measure to assess patients’ condition, the RAID score was initially developed for use in clinical trials and later adopted in clinical practice (22). Recent studies have demonstrated that the RAID score correlates well with the widely used composite measure for RA severity disease activity score 28 (DAS28), and our previous study also showed high responsiveness of RAID score (24, 25). This underscores the RAID score’s reliability as a quantitative tool for measuring health status changes. Our current analysis confirms its strong predictive power, making the RAID score-exclusive model’s performance comparable to that of the model incorporating multiple baseline features. The superior performance of this streamlined model in terms of sensitivity, precision and specificity make it a valuable and user-friendly clinical tool. It effectively identifies patients unlikely to reach remission at 6 months, thus facilitating more effective tailoring of management strategies.

A critical consideration is the false positive rate, which poses a risk of overtreatment for patients who might otherwise achieve remission. In the simulation study for predicting non-remission, by setting a high classification threshold, we significantly enhanced the precision of true non-remission case identification, with a marked reduction in false positives. For instance, under the CDAI criteria, precise early identification with a threshold of 0.81 distinguished 42 patients as non-remission out of 133 true non-remission cases, with only 2 misclassifications (Supplementary Figure S1). This enables timely intervention with alternative treatments and more rigorous monitoring for these identified non-remission patients, potentially increasing the overall remission rate. However, the trade-off for such a high-threshold strategy-a reduced sensitivity-should be also fully considered. Low sensitivity results in failing to detect the majority of non-remission cases, leading to undertreatment and negatively affecting patient outcomes. To mitigate this, lower thresholds that prioritize higher sensitivity could complement the high-threshold approach by capturing a broader range of potential non-remission cases in advance. Defining multiple thresholds tailored to different clinical objectives, as illustrated in Figure 5, can help balance sensitivity and precision, mitigating the risk of both overtreatment and undertreatment in critical practice.

The strong predictive power of RAID score might be attributed to its comprehensive coverage of seven domains of patient-reported outcomes, which is revealed by the impressive performance of simplified model that includes features, such as fatigue as well as PROMIS-PF indicating physical function. Beyond RAID score, this study verifies the predictive value of other baseline characteristics. Notably, PROMIS-PF, which is a health assessment questionnaire (HAQ) was identified as the notable predictor for ACR/EULAR Boolean non-remission. A higher PROMIS-PF, reflecting better physical condition, is inversely associated with non-remission. This finding aligns with previous study demonstrating that the greater disability indicated by higher HAQ was significantly associated with lower likelihood of remission (26). This conclusion is further supported by response outcome prediction models developed by Duong and Gosselt, respectively (17, 27). By contrast, for SDAI and CDAI criteria, fatigue, which has not been fully explored in recent studies, emerged as a significant predictor, particularly for CDAI criteria, revealing its predictive potential. Moreover, the RAI, measuring tender joint count was highlighted as a significant predictor for ACR/EULAR Boolean and SDAI criteria, is in line with previous studies (28). The DAS, identified as a significant predictor for DAS-based EULAR response in earlier research, also demonstrated predictive value in the current study, albeit with moderate predictive power, possibly due to its strong correlation with the RAID score (15, 18). Additionally, factors such as smoking status, sex and ACPA were not identified as top predictors in the present study aligning with negative conclusions of previous study (26).

A strength of this study was that all included patients were DMARD-naïve and initiated methotrexate as the first-line therapy, with data collection conducted in accordance with Good Clinical Practice including independent monitoring, ensuring high data quality (19). Employing an ensemble super learner model, we enhanced performance and mitigated model selection bias. Additionally, the model evaluation on the independent hold-out test set through nested cross-validation ensure robustness, further strengthening the study. Despite this, external validation in other cohorts is needed to address potential overestimation of model performance, and hence remains a limitation. Further validation in cohorts with different ethnicities and clinical context is essential to validate model generalization before broader clinical integration. Moreover, despite promising AUC-ROC, unavoidable false positives necessitate enhanced precision, potentially achievable through a larger sample size. Other limitations may include a restricted patient number and potential limitations with respect to the examined baseline characteristics.

In conclusion, our study provides convincing evidence of the utility of baseline RAID score in predicting non-remission according to ACR/EULAR Boolean, SDAI, CDAI criteria as outcome measures at 6 months. Accordingly, a high baseline RAID score in DMARD-naïve early RA patients starting methotrexate monotherapy should serve as a critical indicator for clinicians. This ‘red flag’ necessitates further evaluation by the proposed model, warranting stringent monitoring to optimize treatment outcomes.

## Data Availability

The original contributions presented in the study are included in the article/Supplementary material, further inquiries can be directed to the corresponding author.
