# Supplementary material for: Development of machine learning models for predicting non-remission in early RA highlights the robust predictive importance of the RAID score-evidence from the ARCTIC study
Source: Front Med (Lausanne). 2025 Feb 12;12:1526708. doi: 10.3389/fmed.2025.1526708 (PMC11861184; doi:10.3389/fmed.2025.1526708)
Supplement: Supplementary file 1 [file Data_Sheet_1.pdf]

Supplementary Table S1. Baseline characteristics in the 222 patients

	Median [25th-75th] or category: Number (%)*	Missing counts (%)
Age, years	53.9 [42.0-62.6]	0 (0)
Sex*	Female: 137 (62%), Male: 85 (38%)	0 (0)
BMI, kg/m^2	25.5 [22.9-28.2]	2 (0.9)
Smoking*	Never: 75 (34%), Ever: 93 (42%), Present: 54 (24%)	0 (0)
Disease duration, months	5.45 [2.8-9.9]	0 (0)
ACPA*	Positive: 182 (82%), Negative: 40 (18%)	0 (0)
RF*	Positive: 157 (71%), Negative: 65 (29%)	0 (0)
DAS	3.3 [2.6-4.2]	0 (0)
RAI	7.0 [4.0-12.8]	0 (0)
SJC44	9.5 [4.0-14.8]	0 (0)
PGA, VAS 0-100mm	49.0 [31.0-69.8]	0 (0)
PhGA, VAS 0-100mm	36.0 [23.0-55.0]	1 (0.5)
ESR, mm/h	19.0 [11.0-30.8]	1 (0.5)
CRP, mg/L	7.0 [3.0-18.0]	0 (0)
Joint pain, VAS 0-100mm	46.5 [29.0-70.0]	0 (0)
Fatigue, VAS 0-100mm	38.0 [14.2-64.8]	0 (0)
PROMIS-PF	39.6 [32.9- 44.2]	0 (0)
CDAI	21.2 [14.8-30.9]	1 (0.5)
SDAI	22.2 [15.7-33.2]	1 (0.5)
RAID score	4.4 [2.9-6.0]	2 (0.9)
Methotrexate starting dose, mg/week	15.0 [15.0-15.0]	0 (0)

Values are median [25th-75th percentile] unless indicated as number (%) by an asterisk (*) for categorical characteristics including Sex (male/female), Smoking (never/ever/present), ACPA (positive/negative) and RF (positive/negative). DAS = Disease Activity Score based on ESR, patient global, SJC 44 and Ritchie Articular Index. ACR = American College of Rheumatology, EULAR = The European Alliance of Associations for Rheumatology, Disease duration = Time since patient reported first swollen joint measured as months, PROMIS-PF = Patient-Reported Outcome Measurement Information Score short form v1.0-physical function 20a T-score with a mean of 50 and standard deviation of 10 in a reference population. ACPA = anti-cyclic citrullinated peptide, RF = Rheumatoid Factor, BMI = Body Mass Index, SDAI = Simplified Disease Activity Index, CDAI = Clinical Disease Activity Index, SJC44 = Swollen Joint Count in 44 joints, VAS = Visual Analogue Scale, PGA = Patients Global Assessment of disease activity, PhGA = Physician Global Assessment of disease activity, RAI = Tender joint count (Ritchie Articular Index, score 0-78), ESR = Erythrocyte Sedimentation Rate, CRP = C Reactive Protein, RAID = Rheumatoid Arthritis Impact of Disease.

Supplementary Table S2. Definition and statistics of predicted clinical outcomes used in the analyses

Treatment outcomes	Count, Proportion	Definition
ACR/EULAR Boolean non-remission at 6 months	143, 64%	The patient must comply with all:<br>• RAI <= 1<br>• SJC28 <= 1<br>• CRP <= 1 mg/dl<br>• PGA <= 1<br>in order to achieve ACR/EULAR remission otherwise non-remission
SDAI non-remission at 6 months	134, 60%	SDAI = TJC28 + SJC28 + PGA/10 + PhGA/10 + CRP/10<br>Remission: < = 3.3 Otherwise non-remission
CDAI non-remission at 6 months	135, 61%	CDAI = TJC28 + SJC28 + PGA/10 + PhGA/10<br>Remission: < = 2.8 Otherwise non-remission

ACR = American College of Rheumatology, EULAR = The European Alliance of Associations for Rheumatology, SDAI = Simplified Disease Activity Index, CDAI = Clinical Disease Activity Index, RAI= Tender joint count (Ritchie Articular Index, score 0-78), SJC28 = Swollen Joint Count in 28 joints, SJC44 = Swollen Joint Count in 44 joints , CRP = C Reactive Protein, PGA = Patients Global Assessment of disease activity, PhGA = Physician Global Assessment of disease activity, TJC28 = Tender Joint Count in 28 joints.

Supplementary Table S3: Statistics of characteristics of patients grouped as remission and non-remission based on specified criteria.

	ACR/EULAR Boolean non-remission at 6 months		SDAI non-remission at 6 months		CDAI non-remission at 6 months	
**Characteristics**	**Remission (36%)**	**Non-remission (64%)**	**Remission (40%)**	**Non-remission (60%)**	**Remission (39%)**	**Non-remission (61%)**
Age, years	51.4 [38.5-63.0]	54.7 [43.5-62.5]	52.4 [39.8-61.8]	54.9 [44.0-62.7]	51.9 [39.6-62.6]	55.0 [44.2-62.5]
Sex*						
-Female	52 (65.8%)	85 (59.4%)	55 (62.5%)	82 (61.2%)	55 (63.2%)	82 (60.7%)
-Male	27 (34.2%)	58 (40.6%)	33 (37.5%)	52 (38.8%)	32 (36.8%)	53 (39.3%)
BMI, kg/m2	24.9 [21.9-27.9]	25.7 [23.5-28.4]	25.0 [22.0-27.5]	25.7 [23.4-28.6]	25.2 [22.0-27.5]	25.5 [23.0-28.6]
Smoking*						
-Never	26 (32.9%)	49 (34.3%)	30 (34.1%)	45 (33.6%)	32 (36.8%)	43 (31.9%)
-Ever	32 (40.5%)	61 (42.7%)	34 (38.6%)	59 (44.0%)	31 (35.6%)	62 (45.9%)
- Present	21 (26.6%)	33 (23.1%)	24 (27.3%)	30 (22.4%)	24 (27.6%)	30 (22.2%)
Disease duration, months	5.4 [3.2-10.7]	5.5 [2.7-9.8]	6.1 [3.2-10.9]	5.0 [2.7-9.7]	6.1 [3.1-10.7]	5.4 [2.7-9.8]
ACPA*						
-Positive	64 (81.0%)	118 (82.5%)	71 (80.7%)	111 (82.8%)	70 (80.5%)	112 (83.0%)
-Negative	15 (19.0%)	25 (17.5%)	17 (19.3%)	23 (17.2%)	17 (19.5%)	23 (17.0%)
RF*						
-Positive	52 (65.8%)	105 (73.4%)	59 (67.0%)	98 (73.1%)	58 (66.7%)	99 (73.3%)
-Negative	27 (34.2%)	38 (26.6%)	29 (33.0%)	36 (26.9%)	29 (33.3%)	36 (26.7%)
DAS	2.9 [2.3-3.5]	3.6 [2.9-4.5]	2.9 [2.3-3.5]	3.7 [2.9-4.5]	3.0 [2.3-3.5]	3.6 [2.8-4.5]
RAI	4.0 [2.5-8.0]	8.0 [5.0-15.0]	4.5 [3.0-8.0]	9.0 [5.0-14.8]	5.0 [3.0-9.0]	8.0 [4.5-14.0]
SJC44	8.0 [4.0-13.0]	10.0 [5.0-15.0]	9.0 [4.0-13.0]	10.0 [5.0-15.8]	10.0 [4.0-13.5]	9.0 [5.0-15.0]
PGA, VAS 0-100mm	34.0 [21.0-52.5]	54.0 [37.5-75.0]	34.5 [21.8-53.0]	55.0 [38.0-76.0]	35.0 [21.0-53.0]	54.0 [37.5-76.5]
PhGA, VAS 0-100mm	30.0 [19.5-42.0]	41.5 [28.0-61.8]	31.0 [20.0-42.5]	42.5 [27.2-63.5]	31.5 [20.0-45.0]	41.0 [26.5-60.5]
ESR, mm/h	16.5 [11.0-27.0]	21.0 [12.0-33.5]	16.0 [10.0-27.0]	21.5 [12.2-35.8]	17.0 [10.0-27.8]	21.0 [12.0-33.5]
CRP, mg/L	5.0 [2.4-10.0]	8.9 [4.0-19.0]	5.0 [2.9-10.5]	9.0 [4.0-21.8]	6.0 [3.0-14.0]	8.0 [4.0-19.0]
Joint pain, VAS 0-100mm	33.0 [21.0-50.5]	53.0 [33.0-73.0]	34.0 [22.8-51.2]	55.0 [33.2-74.0]	34.0 [21.0-51.5]	55.0 [33.5-74.0]
Fatigue, VAS 0-100mm	27.0 [6.5-46.5]	48.0 [24.5-69.0]	27.5 [4.0-46.2]	49.0 [25.0-69.8]	26.0 [3.5-46.0]	50.0 [25.0-70.5]
PROMIS-PF	43.1 [37.3-47.8]	36.1 [30.5-41.6]	42.6 [37.6-46.6]	35.3 [30.1-42.1]	42.6 [37.3-46.6]	35.7 [30.3-42.1]
CDAI	17.3 [9.6-24.2]	24.2 [17.2-34.2]	17.4 [10.3-25.2]	24.4 [17.2-34.3]	17.5 [10.2-26.1]	24.2 [16.6-34.1]
SDAI	18.1 [10.0-25.0]	25.9 [17.8-36.5]	18.4 [11.4-25.9]	26.1 [17.7-36.7]	18.8 [11.4-26.6]	24.9 [17.4-36.4]
RAID score	2.9 [2.0-4.4]	5.3 [3.9-6.6]	3.0 [2.0-4.5]	5.3 [4.0-6.7]	3.0 [2.0-4.4]	5.3 [4.0-6.8]

Methotrexate starting dose, mg/week	15.0 [15.0-15.0]	15.0 [15.0-15.0]	15.0 [15.0-15.0]	15.0 [15.0-15.0]	15.0 [15.0-15.0]	15.0 [15.0-15.0]

Baseline clinical characteristics of the included patients are presented for both groups. Continuous variables are reported as the median [25th–75th percentile], whereas categorical variables, marked with an asterisk (*), are presented as n (percentage).

Supplementary Table S4: Hyperparameters for grid search strategy to optimize prediction models.

Base algorithms	Hyperparameters for Grid Search
Elastic Net	• 'C': [0.001, 0.01, 0.1, 0.5, 1, 5, 10, 100]<br>• 'l1_ratio': [0.001,0.01, 0.1, 0.3, 0.5, 0.7, 0.9, 0.999],<br>• Other hyperparameters: 'penalty': 'elastic net', 'solver': 'saga', 'max_iter': 5000.
Random Forest	• 'max_depth': [5, 7, 10],<br>• 'n_estimators': [75, 100, 125, 150]<br>• 'min_samples_split': [3, 4]<br>• 'max_features' : ['sqrt']
Support vector machine	• 'gamma': ['scale', 'auto']<br>• 'kernel': ['linear', 'poly', 'sigmoid']<br>• 'C': [0.001, 0.01, 0.1, 0.5, 1, 10]

Supplementary Table S5. Performance evaluation across five trials.

	ACR/EULAR non-Remission	SDAI non-Remission	CDAI non-Remission
**Trial 1**	0.75(0.08)	0.77(0.02)	0.76(0.06)
**Trial 2**	0.73(0.06)	0.75(0.12)	0.77(0.05)
**Trial 3**	0.75(0.03)	0.77(0.04)	0.75(0.07)
**Trial 4**	0.77(0.07)	0.73(0.05)	0.75(0.04)
**Trial 5**	0.72(0.11)	0.76(0.04)	0.76(0.04)

The AUC-ROC metrics for each trial presented as the mean with the standard deviation are displayed. AUC-ROC = The area under the receiver operating characteristic curve.

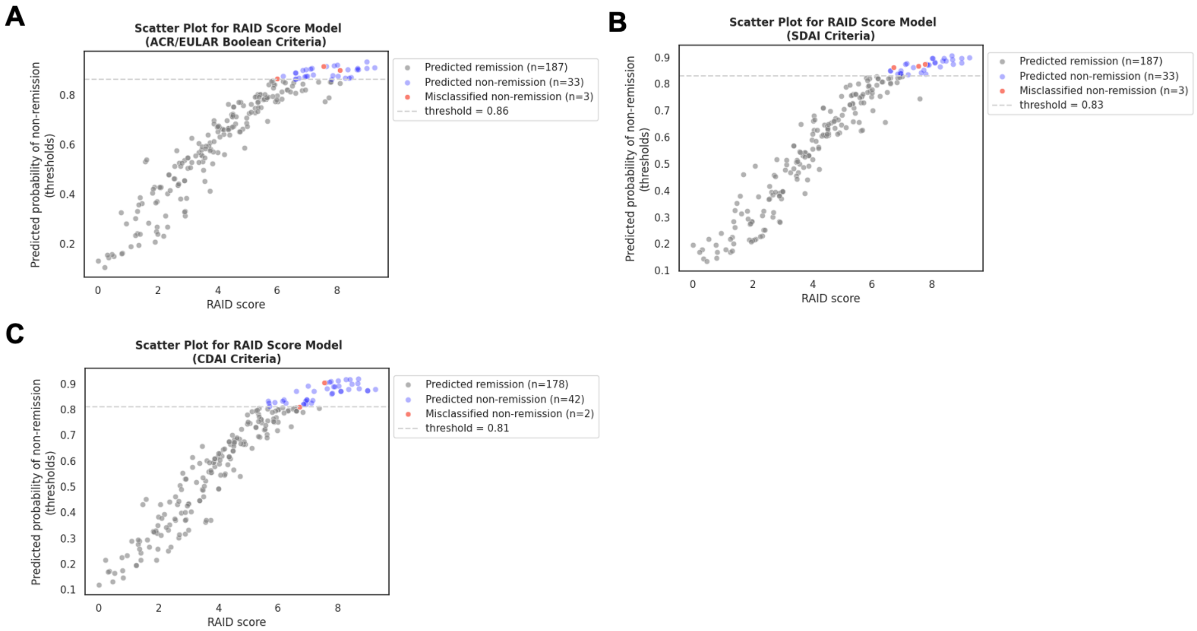

Supplementary Figure S1: Association between RAID score and predicted probability of non-remission. The scatter plot illustrates the relationship between RAID score and predicted probabilities of non-remission based on (**A**) ACR/EULAR Boolean-, (**B**) SDAI-, (**C**) CDAI-criteria using a simplified model that exclusively incorporates the RAID score. Strict thresholds of 0.86, 0.83 and 0.81, identified earlier, are shown with a dashed line respective to each criterion. Blue dots represent predicted non-remission, with misclassified non-remission marked in red.

Supplementary Data

Supplementary Data S1. Data imputation for missing outcomes of ACR/EULAR-, SDAI, CDAI-remission status.

Among the 222 patients included in the study, 12 patients had missing outcomes for ACR/EULAR- and CDAI-remission status. On the other hand, SDAI-remission status was missing for 13 patients, and 16 patients had missing outcomes for DAS-based EULAR non-response. To address these missing outcome variables, data imputation was performed using the following rules; for non-remission outcomes at 6 months: non-remission was determined for patients with missing follow-up visits between 6 and 24 months. Remission was determined if the DAS score was < 1.2 and the patient was on methotrexate monotherapy (including standard and optimized doses) at the 4- and 12-month visits. Patients who continued to receive methotrexate monotherapy (including standard and optimized doses) from 12 to 24 months were considered to be in remission.

Supplementary Data S2. Hyperparameter tuning using grid search strategy and cross-validation.

Hyperparameter tuning is a crucial part of model training process. This was conducted on the model development folds, comprising 80% of total dataset, which has been segmented through nested cross-validation. To achieve the optimal hyperparameter of each base model, grid search strategy combined with cross-validation was used to optimize each model's performance. The grid search aimed to maximize the AUC-ROC by evaluating various combinations of hyperparameters (refer to supplementary Table 4), and 5-fold cross-validation within the grid search process was employed to improve the model generalization. Subsequently, models configured with optimal hyperparameters were initialized for super learner modeling.
